# Comparison Between Transoral Endoscopic Thyroidectomy Vestibular Approach (TOETVA) and Conventional Open Thyroidectomy for Patients Undergoing Total Thyroidectomy and Central Neck Dissection: A Propensity Score-Matching Analysis

**DOI:** 10.3389/fonc.2022.856021

**Published:** 2022-03-02

**Authors:** Haiqing Sun, Xiaojie Wang, Guibin Zheng, Guochang Wu, Qingdong Zeng, Haitao Zheng

**Affiliations:** ^1^Department of Thyroid Surgery, The Affiliated Yantai Yuhuangding Hospital of Qingdao University, Yantai, China; ^2^Department of Thyroid Surgery, Qilu Hospital of Shandong University, Jinan, China

**Keywords:** endoscopic thyroidectomy, safety, surgical completeness, non-stimulated thyroglobulin, vestibular approach

## Abstract

**Background:**

Use of the novel transoral endoscopic thyroidectomy vestibular approach (TOETVA) is increasing worldwide. Although several studies have compared safety and efficacy of TOETVA and other approaches, most focused on comparisons in the context of unilateral thyroidectomy. Therefore, the present study aimed to compare the safety and surgical completeness of TOETVA with conventional open thyroidectomy (COT) in patients with papillary thyroid carcinoma (PTC) undergoing total thyroidectomy and central neck dissection.

**Methods:**

The medical records of patients who underwent TOETVA or COT by a single surgeon between June 2017 and October 2021 were retrospectively reviewed. All patients were diagnosed with PTC and underwent total thyroidectomy with central neck dissection. Propensity score-matching (PSM) was used to reduce potential selection bias and to adjust for differences in baseline clinicopathological characteristics.

**Results:**

After PSM, 84 (TOETVA: 28; COT: 56) patients remained in the study population. There were no significant differences in sex, mean age, combined thyroiditis, tumor size, capsule invasion, tumor multifocality in the same lobe, or tumor location between the groups. Operative time was longer (190.54 ± 28.26 *vs.* 123.93 ± 29.78 min, *P*<0.001), while postoperative drainage volume (161.07 ± 225.30 *vs.* 71.16 ± 28.56 ml, *P*=0.045) was greater, in the TOETVA group than in the COT group. The groups exhibited no significant differences in the mean number of central lymph nodes retrieved (9.39 ± 4.01 *vs.* 10.71 ± 5.17, *P*=0.202), mean number of metastatic central lymph nodes (1.36 ± 1.93 *vs.* 1.77 ± 2.31, *P*=0.421), postoperative mean thyroglobulin levels (0.08 ± 0.24 *vs.* 0.10 ± 0.27, *P*=0.686), rate of transient hypoparathyroidism (TOETVA: 67.9% *vs.* COT: 66.1%, *P*=0.870), rate of transient vocal cord palsy (TOETVA: 0% *vs.* COT: 1.8%, *P*=1.000), or other complications (TOETVA: 3.6% *vs.* COT: 0%, *P*=0.333).

**Conclusions:**

TOETVA is a safe approach in select patients with PTC and exhibits similar efficacy to COT in terms of surgical completeness.

## Introduction

Following the first transoral thyroidectomy performed by Wilhelm and Metzig in 2009 (1), Nakajo et al. and Wang et al. reported the first use of the transoral endoscopic thyroidectomy vestibular approach (TOETVA) in 2013 (2, 3), which is now widely utilized worldwide. Richmon et al. reported the first experience of transoral robotic-assisted thyroidectomy in two cadavers (4). Transoral robotic thyroidectomy, including both the sublingual and vestibular approaches, was also reported (5–7), which can serve as an expansion of the transoral endoscopic approach. Several recent studies have compared the safety and efficacy of TOETVA with those of other approaches, such as the areolar approach (8), bilateral axillo-breast approach (9), gasless transaxillary approach (10), and conventional open thyroidectomy (COT) (11, 12), confirming that the TOETVA matched other approaches in terms of surgical safety and efficacy and was able to achieve lymph node dissection comparable to COT. However, most patients in these previous studies underwent unilateral thyroidectomy, and comparative studies of total thyroidectomy and central neck dissection are lacking. Furthermore, comparisons with unilateral thyroidectomy are insufficient for evaluating the safety and surgical completeness of TOETVA. Therefore, in the present study, we aimed to compare the safety and surgical completeness of TOETVA with COT in patients with papillary thyroid carcinoma (PTC) who underwent total thyroidectomy and central neck dissection.

## Materials and Methods

This retrospective study was conducted in the Department of Thyroid Surgery at The Affiliated Yantai Yuhuangding Hospital of Qingdao University. The study was approved by the Ethical Committee of The Affiliated Yantai Yuhuangding Hospital of Qingdao University. In our department, TOETVA is indicated in patients with the following characteristics: (a) diagnosis of papillary thyroid carcinoma *via* fine needle aspiration cytology before surgery, with tumor sizes ≤1.5 cm in the upper pole or ≤3 cm in the other parts of the thyroid, including patients with clinically positive central nodes and (b) diagnosis of benign with a tumor size ≤6 cm. The exclusion criteria were as follows: (a) history of neck surgery, (b) history of neck radiation therapy, (c) lateral cervical lymph node metastasis, and (d) tumor invasion to the adjacent organs.

All available thyroidectomy approaches were clearly explained to all included patients using multimedia prior to surgery. Patients were allowed to freely select their preferred approach and provided written informed consent. Only some young women tend to choose TOETVA or endoscopic thyroidectomy *via* the areolar approach due to the need for cosmetic intervention. Men and older women tend to prefer COT because of cost, trauma, and endoscopic-related complications.

A total of 28 patients underwent total thyroidectomy and central neck dissection *via* TOETVA by a single surgeon in our department from July 2017 to October 2021. A total of 597 patients underwent open total thyroidectomy and central neck dissection by the same surgeon during the same period, and 597 patients were included as controls.

The surgical technique of TOETVA has been described in detail in previous articles by our team (10, 13). In summary, the surgery starts with three oral vestibule incisions. Clamp forceps are used to create and dilate the workspace in the submental region. The workspace is maintained using carbon dioxide (CO_2_) at 4–6 mmHg after the trocars are inserted. Electrocautery and an ultrasound scalpel are used to widen the workspace until the sternal notch and bilateral sternocleidomastoid muscles are visible. Total thyroidectomy and central neck dissection are performed in a similar manner with COT. Intraoperative nerve monitoring and indocyanine green fluorescence imaging were not used because they were not available in our hospital. All patients got a drain post-operatively in the submental region.All patients underwent thyroid ultrasonography or computed tomography to exclude suspicious lateral cervical lymph node metastasis or invasion to the adjacent organs. In the TOETVA group, cefuroxime sodium and metronidazole were administered 30 min prior to the induction of anesthesia and continued until 24 h postoperatively. All patients were required to use chlorhexidine gargle for 7 days postoperatively. Patients were discharged from the ward on the first to third postoperative day when they did not have hypocalcemia-related symptoms, without the need for intravenous calcium supplementation.

Venous blood was collected 1 day before surgery to measure parathyroid hormone (PTH) and calcium levels to exclude hyperparathyroidism. Serum PTH levels were measured at 1 day, 1 month, 3 months, and 6 months postoperatively. Serum thyroglobulin (Tg) levels were measured 1 and 3 months after surgery and twice a year thereafter. Neck ultrasound examinations were performed twice a year after surgery.

Medical records were retrospectively reviewed to collect data related to the surgical approach, patient sex and age, maximum tumor diameter, presence of Hashimoto’s thyroiditis, capsule invasion, tumor multifocality in the same lobe, tumor location, operative time (cut-to-suture time), postoperative drainage volume, preoperative and postoperative PTH levels, recurrent laryngeal nerve (RLN) palsy, number of retrieved central lymph nodes, number of metastatic central lymph nodes, and postoperative Tg levels.

Permanent hypoparathyroidism was defined as follows: (a) PTH levels <15 pg/ml or (b) the need for supplementation with calcium and calcitriol for more than 6 months after surgery. Permanent RLN paralysis was defined as nonrecovery of vocal cord function within 6 months.

We assessed the surgical safety of each operative approach by evaluating transient and permanent RLN palsy, transient and permanent hypoparathyroidism, and other complications. We also measured the number of retrieved central lymph nodes, number of metastatic central lymph nodes, and postoperative Tg levels to assess surgical completeness.

### Statistical Analyses

Propensity score-matching (PSM) was performed to minimize patient selection bias and to adjust for differences in baseline clinicopathological characteristics. The propensity scores of the individuals were calculated using logistic regression analysis (Statistical Package for the Social Sciences [SPSS] version 26.0. Armonk, NY). For PSM, seven clinicopathological factors that may have affected the surgical outcomes were selected as covariates, including patient sex, patient age at surgery, presence of Hashimoto’s thyroiditis, diameter of the largest tumor, thyroid capsule invasion, tumor multiplicity in the same lobe, and tumor location. After including all variables above, we performed 1:2 PSM using the nearest neighbor method with a caliper width of 0.06 of the standard deviations of the logit of the propensity score.

Data were analyzed using independent-samples *t*-tests, χ^2^ tests, or Fisher’s exact tests in SPSS software (version 26.0; IBM Corp, Armonk, NY, USA). Statistical significance was set at *P*<0.05.

## Results

### Clinical and Surgical Characteristics

**Figure 1:**
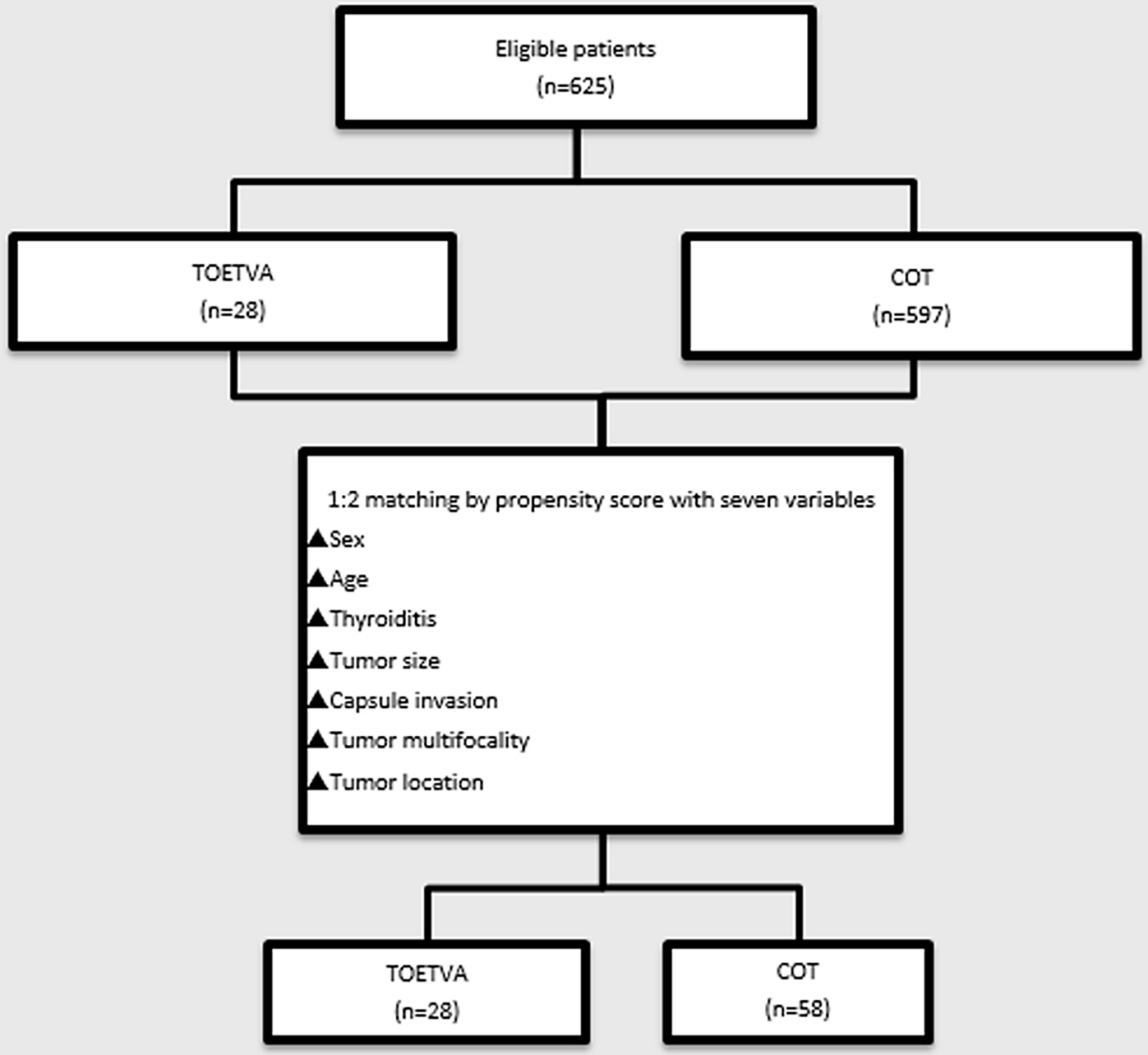
Flowchart of propensity score-matching. TOETVA, transoral endoscopic thyroidectomy vestibular approach; COT, conventional open thyroidectomy.

During the study period, 625 eligible patients underwent total thyroidectomy and central neck dissection, 28 of whom selected TOETVA. The remaining 597 patients selected COT. After PSM, 84 (n=28, TOETVA group; n=56, COT group) patients remained in the study population (**Figure 1**), and the two matched groups were well balanced in terms of the seven covariates. No significant differences were observed between the two groups in terms of sex (P=1.000), mean age (TOETVA, 36.57 ± 8.03 *vs.* COT, 39.66 ± 8.67, P=0.119), presence of combined thyroiditis (P=0.874), tumor size (TOETVA, 1.04 ± 0.55 *vs.* COT, 0.95 ± 0.56 cm, P=0.467), capsule invasion (P=0.620), tumor multiplicity in the same lobe (P=0.278), or tumor location (P=0.430) (**Table 1**). The operative time (cut-to-suture time) was considerably longer in the TOETVA group than in the COT group (190.54 ± 28.26 *vs.* 123.93 ± 29.78 min, respectively, P<0.001. The postoperative drainage volume was higher in the TOETVA group than in the COT group (161.07 ± 225.30 *vs.* 71.16 ± 28.56 ml, P=0.045). There were no significant differences in postoperative hospital stay between the two groups (2.21 ± 1.34 *vs.* 1.88 ± 0.94, P=0.181) (**Table 1**).

**Table 1:** Clinical and surgical characteristics of included patients following propensity score-matching (N=84).

Variables	TOETVA (n=28)	COT (n=56)	*P*
Sex			1.000
Male	1	2	
Female	27	54	
Age (years)	36.57 ± 8.03	39.66 ± 8.67	0.119
Hashimoto’s thyroiditis			0.874
Yes	11	21	
No	17	35	
Diameter of the largest tumor (cm)	1.04 ± 0.55	0.95 ± 0.56	0.467
Thyroid capsule invasion			0.620
Yes	20	37	
No	8	19	
Tumor multifocality in the same lobe			0.278
Yes	13	33	
No	15	23	
Tumor location			0.430
Isthmus	1	0	
Left	2	5	
Right	3	10	
Both	22	41	
Operation time (min)	190.54 ± 28.26	123.93 ± 29.78	<0.001
Postoperative hospital stay (d)	2.21 ± 1.34	1.88 ± 0.94	0.181
Postoperative drainage volume (ml)	161.07 ± 225.30	71.16 ± 28.56	0.045

TOETVA, transoral endoscopic thyroidectomy vestibular approach; COT, conventional open thyroidectomy.

### Surgical Completeness

Surgical completeness according to the number of central lymph nodes retrieved and serum Tg levels are shown in **Table 2**. Neither the mean number of retrieved central lymph nodes (9.39 ± 4.01 *vs.* 10.71 ± 5.17, P=0.202) nor the mean number of metastatic central lymph nodes (1.36 ± 1.93 *vs.* 1.77 ± 2.31, P=0.421) differed significantly between the TOETVA and COT groups. At 3–54 months after surgery, the mean Tg level without thyroid-stimulating hormone (TSH) stimulation was 0.09 ± 0.26 ng/ml, and there was no significant difference between the TOETVA and COT groups (0.08 ± 0.24 *vs.* 0.10 ± 0.27, P=0.686). The percentages of patients with Tg values <0.27 ng/ml were 92.9% and 85.7% in the TOETVA and COT groups, respectively. The percentages of patients with Tg values <0.1 ng/ml were 82.1% and 80.4% in the TOETVA and COT groups, respectively. The percentages of patients with Tg values <0.04 ng/ml were 67.9% and 71.4% in the TOETVA and COT groups, respectively. No significant differences between the groups were observed at any of the Tg cut-off levels (0.27 ng/ml, P=0.551; 0.1 ng/ml, P=0.884; 0.04 ng/ml, P=0.736).

**Table 2:** Surgical completeness (N=84).

Variables	TOETVA (n=28)	COT (n=56)	*P*
Central lymph nodes			
Retrieved	9.39 ± 4.01	10.71 ± 5.17	0.202
Metastatic	1.36 ± 1.93	1.77 ± 2.31	0.421
Tg level (ng/ml)	0.08 ± 0.24	0.10 ± 0.27	0.686
Tg ≥0.27	2 (7.1%)	8 (14.3%)	0.551
Tg <0.27	26 (92.9%)	48 (85.7%)	
Tg ≥0.1	5 (17.9%)	11 (19.6%)	0.844
Tg <0.1	23 (82.1%)	45 (80.4%)	
Tg ≥0.04	9 (32.1%)	16 (28.6%)	0.736
Tg <0.04	19 (67.9%)	40 (71.4%)	

Tg, thyroglobulin.

### Complications

Postoperative complications are shown in **Table 3**. There were no significant differences in the percentage of patients with transient hypoparathyroidism (TOETVA 67.9% *vs.* COT 66.1%, P=0.870) or permanent hypoparathyroidism (TOETVA 0% *vs.* COT 1.8%, P=1.000) between the groups. One patient in the TOETVA group developed vocal cord palsy (3.6%) but recovered within 3 months. No patients in either group developed permanent vocal cord palsy. One patient in the TOETVA group developed chylous fistula (3.6%) and recovered after 8 days of treatment with peripheral total parenteral nutrition and octreotide. One patient in the COT group developed permanent hypoparathyroidism (1.8%). None of the patients in either group experienced postoperative bleeding, seroma, or surgical site infection. Twenty-three patients in the TOETVA group experienced varying degrees of numbness around the jaw area, and 21 of them recovered within 1–3 months after surgery. Two patients remained unrecovered 3 years after surgery and were considered to have permanent mental nerve injury.

**Table 3:** Postoperative complications (N=84).

Variables	TOETVA (n=28)	COT (n=56)	*P*
Hypoparathyroidism			
Transient	19 (67.9%)	37 (66.1%)	0.870
Permanent	0 (0%)	1 (1.8%)	1.000
Vocal cord palsy			
Transient	1 (3.6%)	0 (0%)	0.333
Permanent	0 (0%)	0 (0%)	1.000
Other complications			0.333
Chylous fistula	1 (3.6%)	0 (0%)	
Seroma	0 (0%)	0 (0%)	
Infection	0 (0%)	0 (0%)	
Bleeding	0 (0%)	0 (0%)	
Mental nerve injury			–
Transient	21 (78.57%)	–	
Permanent	2 (7.2%)	–	

### Postoperative Follow-Up

**Figure 2:**
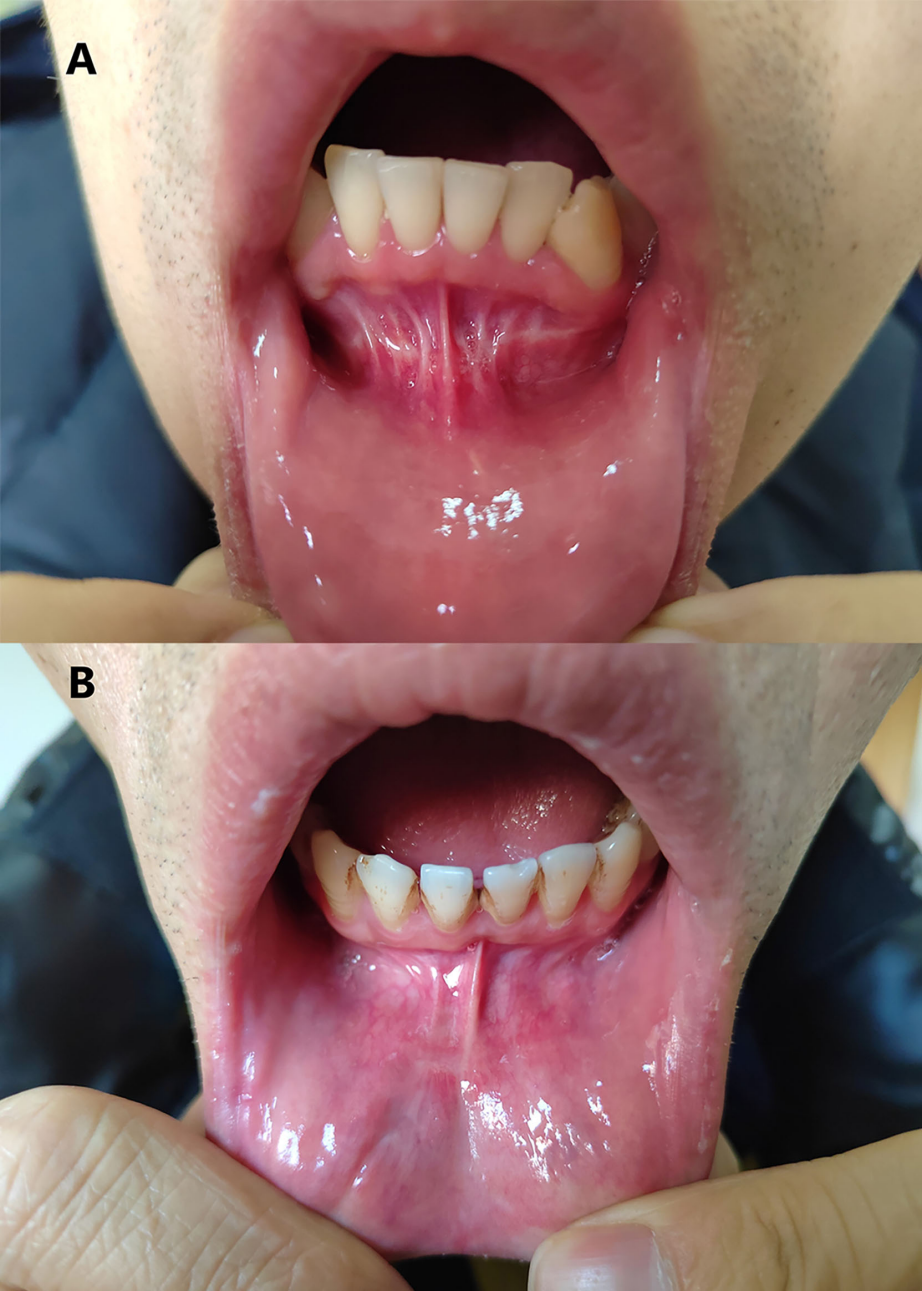
Differences in oral scars after TOETVA for different incision types. **(A)** Oral scar prior to modification of the vestibular incision. **(B)** Oral scar after modification of the vestibular incision.

In both groups, the incisions completely healed within 1 week. Four patients who underwent surgery between June 2017 and July 2018 developed visible synechia with the gingiva and scarring. The other patients only had a light white line after the original middle vestibular straight incision was changed to an arc incision and adjusted the three incisions closer to the lower lip (13) (**Figure 2**). After a median follow-up of 16 months (range, 3–54 months) after surgery, no evidence of recurrence or residual lymph nodes was observed in any patient of either group. There were no significant differences in quality of life between the two groups except that some patients in the TOETVA group complained of numbness around the jaw area before they recovered.

## Discussion

Since Anuwong (14) first reported success for 60 cases in 2016, TOETVA has become increasingly common worldwide. Despite advancements in the procedure during this time, studies regarding the use of TOETVA for total thyroidectomy and central neck dissection remain lacking, possibly due to the time required or concerns regarding complications and surgical completeness. Therefore, this study aimed to evaluate the safety and surgical completeness of TOETVA when compared with COT in patients undergoing total thyroidectomy and central neck dissection.

Following PSM to minimize patient selection bias and differences in confounding factors in multiple analyses, baseline characteristics (including patient sex, patient age at surgery, presence of Hashimoto’s thyroiditis, diameter of the largest tumor, thyroid capsule invasion, tumor multiplicity in the same lobe, and tumor location) were similar between the two groups.

**Figure 3:**
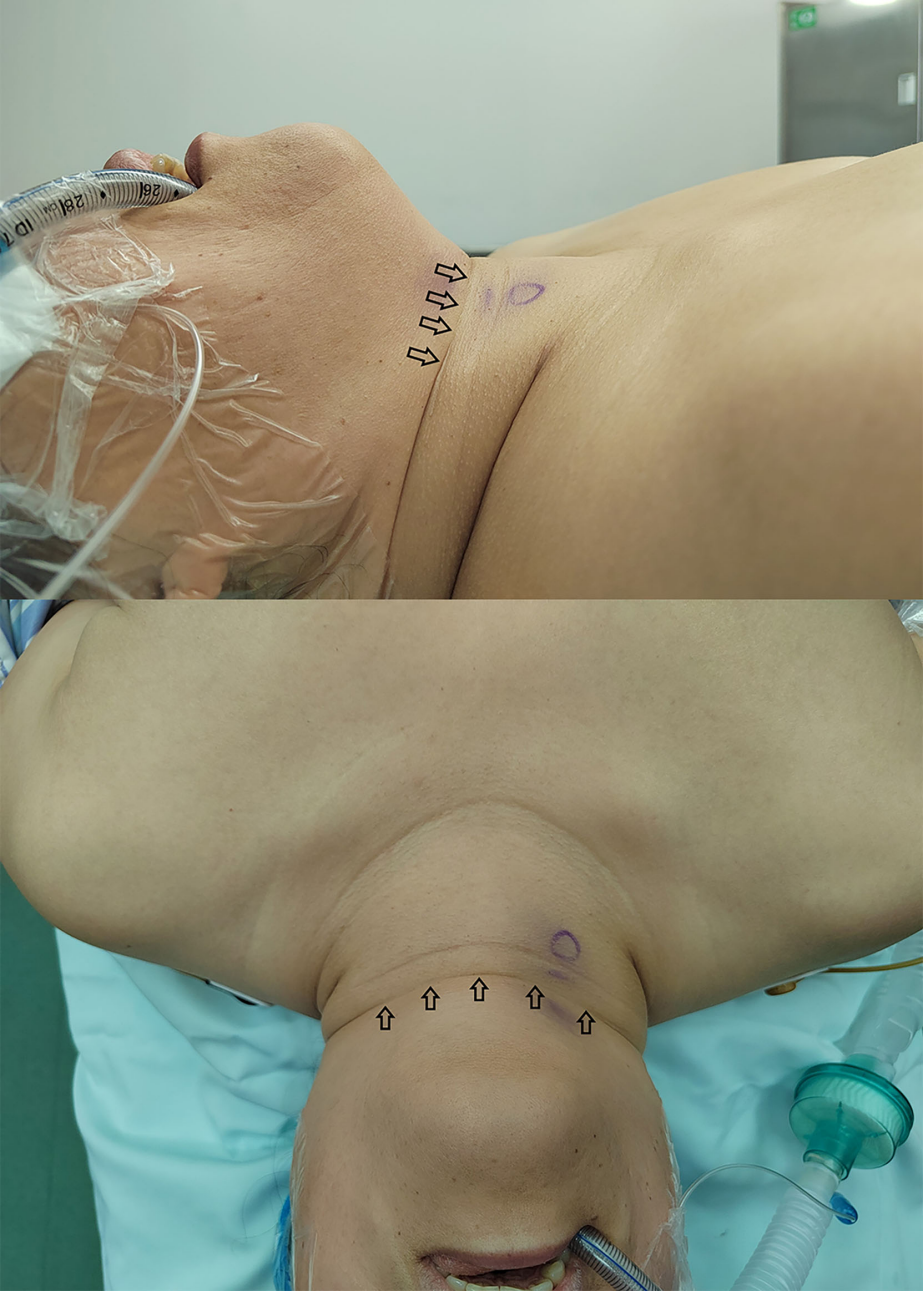
Wrinkle between the submental skin and the skin of the neck.

The operative time was longer for TOETVA than for COT, which is consistent with most previous reports (11, 15–18). This is a common phenomenon in that, for most surgeons, it takes more time to perform the same procedure using an endoscope than *via* an open approach. However, more time is required to create the working space for TOETVA than for COT. Deroide et al. (19) further observed that the workspace was greater in patients with wide necks than in those with slender necks. Smaller workspaces can make the operation more difficult under the endoscope, which can lead to a longer operation time. We observed that the workspace was smaller if the wrinkle between the submental skin and the skin of the neck cannot be unwrinkled completely after the patient was placed in the supine position with the neck extended slightly (**Figure 3**). Our findings indicate that a long and wide mandible, a narrow neck, and diseases affecting the cervical vertebrae are likely to cause difficulty in complete unwinking.

The postoperative drainage volume was higher in the TOETVA group than in the COT group, but that did not cause longer postoperative hospital stay because most patients were discharged with a drainage tube and extubated at a community hospital when the drainage rate was less than 20 ml per day.

**Figure 4:**
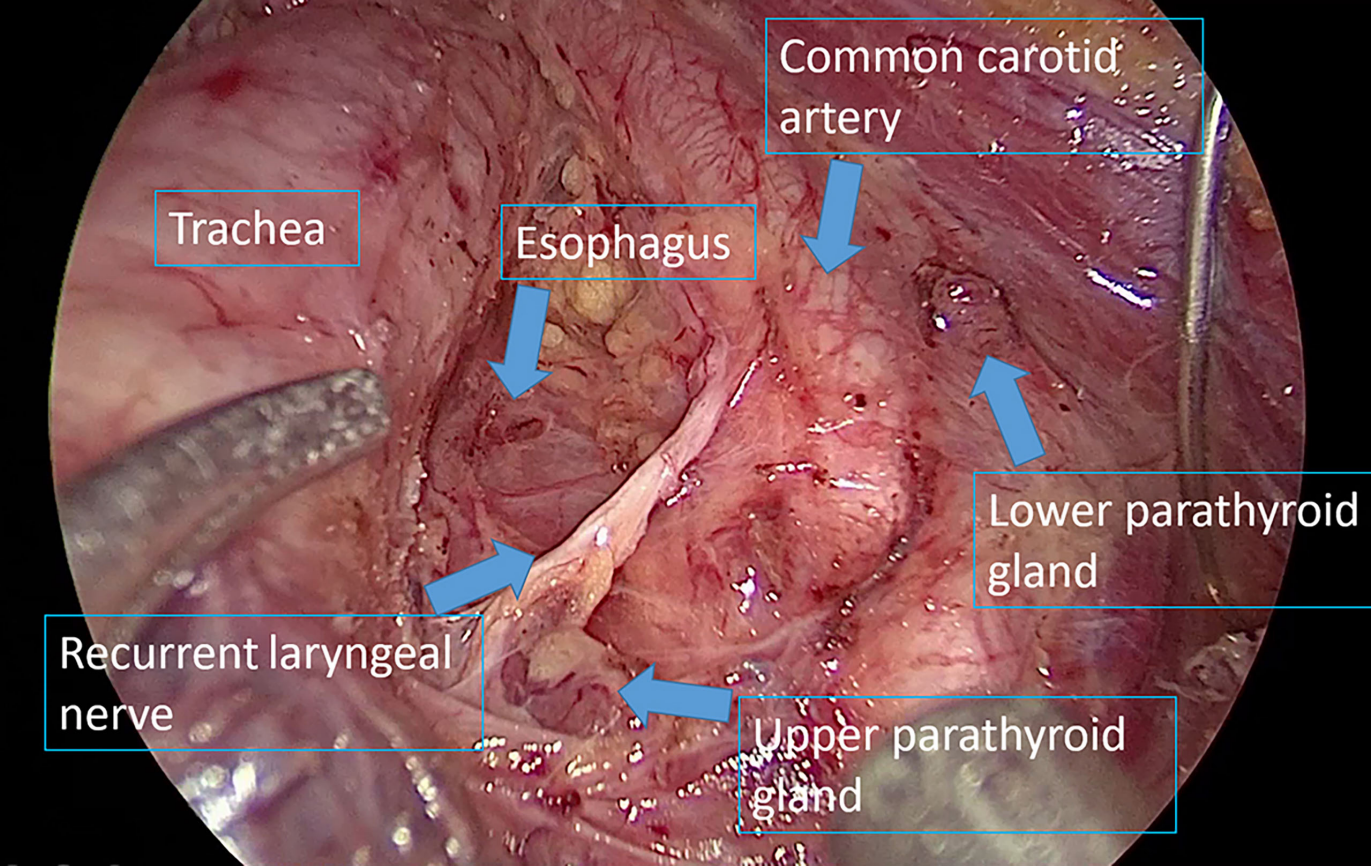
Surgical field after central lymph node dissection.

Surgical completeness was measured based on the number of central lymph nodes retrieved, number of metastatic central lymph nodes, and postoperative Tg levels. We previously reported no differences in these parameters between TOETVA and COT for patients with unilateral thyroid carcinoma undergoing central lymph node dissection (15). In this study, we also observed no differences in bilateral cervical lymph node dissection between the TOETVA and COT groups. The mean numbers of central lymph nodes retrieved were 9.39 ± 4.01 and 10.71 ± 5.17 in the TOETVA and COT groups, respectively, which are higher than the numbers reported by Ahn et al. (16) (4.98 ± 3.12 and 5.70 ± 4.35 in the TOETVA and COT groups, respectively). This may be due to the different preferences of the surgeons in the studies. We routinely resected all lymph nodes in the central region, including those posterior to the RLN, even if the tumor was small or there was no indication of central lymph node enlargement on preoperative ultrasound (**Figure 4**). This is because many reports suggest a high rate of central lymph node metastasis in patients with PTC, even if the tumor size is <1 cm (20, 21). Moreover, Chinese guidelines recommend preventive central lymph node dissection for patients with PTC (22, 23). The mean numbers of metastatic central lymph nodes were 1.36 ± 1.93 and 1.77 ± 2.31 in the TOETVA and COT groups, respectively, which are compatible with the numbers reported by Ahn et al. (16) (1.08 ± 1.46 and 1.70 ± 2.35 in the TOETVA and COT groups, respectively).

Our analysis also indicated that the mean Tg level without TSH stimulation did not significantly differ between the two groups at 3–54 months after surgery. Previous studies reported that the rate of PTC recurrence was low in patients with a non-stimulated Tg level less than 0.27 (24) or 0.1 ng/ml (25), as cited by the 2015 American Thyroid Association guidelines (26). A Tg level <0.04 ng/ml is considered undetectable in our department. We observed no significant differences between the groups based on different Tg cut-off levels (0.27 ng/ml, P=0.551; 0.1 ng/ml, P=0.884; 0.04 ng/ml, P=0.736), indicating that surgical completeness was similar for TOETVA and COT.

Surgical safety was evaluated based on the complication rate. In our study, 67.9% and 66.1% of patients in the TOETVA and COT groups, respectively, developed transient hypoparathyroidism, significantly higher rates than those reported in other studies (16, 17, 27). These rates may have been lower in previous studies due to differences in the definition of hypoparathyroidism, postoperative time points at which PTH was measured, and the proportion of pathological malignancy. In our study, hypoparathyroidism was defined as PTH <15 pg/ml, and venous blood was collected on postoperative day 1 to measure PTH levels. In their study, Anuwong et al. (17) reported a hypoparathyroidism rate of approximately 10.9%, but only 6.2% (26/422) of patients were diagnosed with malignancy, and only 41.9% (177/422) of patients underwent bilateral thyroidectomy. Ahn et al. (16) reported that the rates of transient hypoparathyroidism were 12.5% (4/40) and 24.7% (21/80) in the TOETVA and COT groups, respectively. However, in their study, hypoparathyroidism was defined as PTH <10 pg/ml, and PTH levels were measured in the immediate postoperative period (<2 weeks). You et al. (27) reported transient hypoparathyroidism rates of 16.6% (2/12) and 25% (3/12) in the transoral robotic thyroidectomy and open groups, respectively, although hypoparathyroidism was defined as PTH <8 pg/ml in their study. In our study, one patient (1.8%) in the COT group developed permanent hypoparathyroidism, and there were no significant differences between the two groups in terms of transient hypoparathyroidism or permanent hypoparathyroidism. Furthermore, one (3.6%) patient in the TOETVA group developed transient vocal cord palsy but recovered within 3 months, and no patients in either group developed permanent vocal cord palsy. Several other studies have also reported low rates of permanent hypoparathyroidism and vocal cord palsy following TOETVA (13, 16–18). While chylous fistula occurred in one patient in the TOETVA group, this patient recovered after 8 days of conservative treatment. In the early stage, two patients in the TOETVA group experienced permanent mental nerve injury, which did not recur after we changed the original middle vestibular straight incision to an arc incision and adjusted the three incisions closer to the lower lip, a process described in detail in our previous reports (13, 28). Chai et al. (29) suggested that the midline incision be made 1 cm above the frenulum of the lower lip to allow for greater flexibility of the endoscope. Furthermore, Zhang et al. (30) noted that synechia formation was more likely when the middle vestibular incision was made <1 cm from the gingiva. Our modified incision method also appeared to reduce the rate of mental nerve injury (13, 28). The transoral and submental thyroidectomy approach had been reported to reduce the incidence of mental nerve injuries and is recommended for slightly larger thyroid nodules (31, 32). It can also be utilized as an alternative surgical procedure. Based on these findings, we concluded that the safety of TOETVA is similar to that of COT.

### Limitations

Our study had some limitations. First, as this was a retrospective study, patient selection bias and confounding differences still existed between the two groups, although the PSM method was used to ensure balance in terms of seven baseline characteristics. Second, due to concerns regarding complications related to the RLN and parathyroid glands, patients with unilateral disease were more likely to select TOETVA in the early stage. As of October 2021, 403 patients had undergone TOETVA in our department, only 29 of whom underwent total thyroidectomy (one patient was excluded due to a pathologically benign diagnosis); therefore, the sample size of this study was insufficiently large. Third, synthetic TSH is not available in China. As stopping levothyroxine is not conducive to the treatment of PTC, only non-stimulated Tg levels were measured in this study. Fourth, postoperative laryngoscopy is only performed when the patients or doctors sense abnormalities in sound, which can lead to underestimation of RLN injury. Lastly, the follow-up period was insufficiently long to observe tumor recurrence.

## Conclusions

Our study focused on the safety and surgical completeness of TOETVA in patients with PTC who require total thyroidectomy plus central neck dissection, which has rarely been investigated in previous studies. The current results suggest that TOETVA can be safely performed in select patients with PTC and is similar to COT in terms of efficacy/surgical completeness. Thus, our study provides more evidence for the oncological safety of TOETVA in patients with PTC than previous studies, which primarily focused on unilateral thyroidectomy. Nonetheless, studies with larger sample sizes and longer follow-up periods are required to examine tumor recurrence following TOETVA.

## Data Availability Statement

The original contributions presented in the study are included in the article/**Supplementary Material**. Further inquiries can be directed to the corresponding author.

## Ethics Statement

The studies involving human participants were reviewed and approved by the Ethical Committee of The Affiliated Yantai Yuhuangding Hospital of Qingdao University. Patients provided their written informed consent to participate.

## Author Contributions

Study design: HS, HZ, and QZ. Data collection: HS and XW. Data analysis: HS, GW, and GZ. Drafting the manuscript: HS. Project supervision: HZ. All authors contributed to the article and approved the submitted version.

## Funding

Science and technology project of Shandong Society of Geriatrics (LKJGG2021W128).

## Conflict of Interest

The authors declare that the research was conducted in the absence of any commercial or financial relationships that could be construed as a potential conflict of interest.

## Publisher’s Note

All claims expressed in this article are solely those of the authors and do not necessarily represent those of their affiliated organizations, or those of the publisher, the editors and the reviewers. Any product that may be evaluated in this article, or claim that may be made by its manufacturer, is not guaranteed or endorsed by the publisher.
